# Transdiagnostic determinants of access to mental health care for youth with disabilities: a systems-oriented structured narrative review

**DOI:** 10.3389/fpubh.2026.1782003

**Published:** 2026-05-12

**Authors:** Romina Rinaldi, Maëlle Neveu, Filip Morisse

**Affiliations:** 1 Department of Clinical Orthopedagogy, University of Mons, Mons, Belgium; 2 Center for Interdisciplinary Research on Disability and Inclusion, University of Mons, Mons, Belgium; 3 EQUALITY//ResearchCollective, Universiteit Gent Vakgroep Orthopedagogiek, Ghent, Belgium

**Keywords:** access to mental health care, children and youth, disability, health equity, public mental health

## Abstract

**Objective:**

Children and young people with disabilities are at increased risk of emotional and behavioral difficulties and mental health problems, yet determinants shaping access to mental health care remain insufficiently understood.

**Methods:**

A structured search of PubMed, ScienceDirect, and Scopus was conducted in March 2024. A total of 228 publications published between 1999 and 2024 were analyzed using thematic analysis to identify key determinants influencing access to mental health care.

**Results:**

Thematic analysis identified five cross-cutting dimensions, emerging inductively from the literature and informed by disability studies, child development, and health equity frameworks: (1) fragmented and poorly coordinated service systems; (2) barriers to accessibility and equity; (3) limited adequacy of care due to diagnostic overshadowing and training gaps; (4) restricted, often tokenistic participation of young people and families; and (5) stigma embedded within professional and structural practices.

**Conclusion:**

Findings highlight the need to move beyond deficit-oriented models toward a universal design for health care emphasizing participation, emotional safety, and continuity to reduce inequities, improve quality of life, and create equitable, sustainable care.

## 1 Background

Children and young people with disabilities constitute a heterogeneous population characterized by significant and enduring functional limitations arising from neurodevelopmental disorders and chronic health conditions. These limitations may affect mobility, communication, learning, cognition, and sensory processing and are typically associated with substantial support needs across multiple domains of daily life.

Far from being inconsequential, these lived realities exert a direct influence on the mental health of young people with disabilities (1–4). This group is at a greater risk of mental disorders than their typically developing peers, particularly with respect to anxiety, depression, and behavioral difficulties [e.g., (5–8)]. However, their mental health needs remain insufficiently recognized in both research and service provision.

A central reason for this under-recognition lies in how symptoms are presented and interpreted. Difficulties often manifest atypically, particularly in young people with limited verbal expression or complex developmental profiles, making identification and diagnosis challenging (3, 9–14). For example, emotional distress may manifest through behavioral changes such as irritability, withdrawal, or outbursts rather than through verbal reports of sadness or anxiety. These challenges highlight the limitations of the deficit-oriented and narrowly medical approaches. Contemporary conceptual models, such as the International Classification of Functioning, Disability, and Health (15) and the Disability Creation Process (16), overcome these limitations by emphasizing the dynamic interplay between functional limitations and social, institutional, and physical environments. They invite a shift toward perspectives centered on participation, agency, and social determinants of inclusion, which are essential for understanding and addressing the mental health of young people with disabilities. From this perspective, the consequences of mental-health difficulties cannot be examined solely at the level of individual symptoms but must also be considered in relation to the accessibility of environments, the availability of adapted supports, and the quality of social and institutional responses.

The consequences of unmet mental health needs must therefore be understood within a broader ecological and participatory perspective. Difficulties interact with limitations in communication, autonomy, and participation, resulting in long-term restrictions on development and quality of life. Quality of life in this population is shaped by physical health and functional capacity, as well as by the accessibility of the environment, the availability of adapted support, and the quality of relationships (17, 18). Untreated or misdiagnosed mental health conditions further erode QoL, reinforcing cycles of exclusion and dependency (1, 5, 10, 19, 20). Families also experience significant strain with high levels of stress, disrupted routines, and caregiver fatigue (21, 22). Conversely, poor QoL, especially when characterized by relational deprivation and a lack of meaningful engagement, increases the risk of psychological distress (23–25).

These realities contradict international human rights frameworks. The United Nations Convention on the Rights of Persons with Disabilities (CRPD) (26) recognizes the rights of children and young people with disabilities to the highest attainable standard of health, including mental health, without discrimination. This right encompasses access to timely, tailored, and responsive care that addresses communication needs, developmental trajectories, and family context. Meeting this challenge requires acknowledging the diversity of disability-related needs and the existence of common obstacles that cut across diagnostic categories. Young people with disabilities often encounter recurring barriers in their care trajectories, regardless of specific conditions. Recognizing these shared dimensions is essential for developing approaches that move beyond categorical frameworks and support more coherent and inclusive systems of care. However, translating these principles into practice remains challenging. Despite the increasing recognition of mental health needs among young people with disabilities, the existing literature does not yet provide an integrated picture of how access is organized and achieved. Most studies remain narrowly focused and often examine specific diagnostic groups or individual support. This segmented approach makes it difficult to identify broader, cross-cutting determinants of inclusion and exclusion and limits the development of coherent strategies to improve access. To overcome these limitations, it is necessary to move beyond siloed approaches and adopt a transdiagnostic perspective that focuses on common cross-cutting concerns rather than diagnostic differences, to capture mechanisms shared across disability contexts and to guide the design of more inclusive care systems.

Despite growing recognition of the mental health needs of children and young people with disabilities, existing research remains highly fragmented across diagnostic categories, service sectors, and disciplinary traditions. This fragmentation limits the identification of shared mechanisms shaping inequities in access to care and service adequacy.

This review addresses the following research question:

*Which cross-cutting, system-level determinants shape inequities in access to mental health care for children and young people with disabilities across diagnostic groups?*

In this review, access to mental health care refers to the ability of children and young people with disabilities and their families to identify mental health needs, seek appropriate support, and obtain timely and effective care. Building on this conceptualization and adopting a transdiagnostic public mental health perspective, the aim of this review is to synthesize evidence on shared structural and relational mechanisms that transcend diagnostic boundaries and to inform the design of more inclusive, equitable, and developmentally responsive mental health care systems.

## 2 Methods

### 2.1 Aims

This review had three objectives: (a) identifying recurring structural and systemic barriers to mental health care for young people with disabilities; (b) mapping support needs and promising practices across conditions and contexts; and (c) highlighting strategies for organizing services that promote greater equity, continuity, and developmental responsiveness.

### 2.2 Study design

The research question guiding this review required an approach capable of integrating diverse forms of knowledge and addressing complexity across multiple levels of analysis. Because the objective was to understand how structural, organizational, and relational mechanisms intersect to shape access to mental health care for children and young people with disabilities, a structured narrative review design was selected. This approach enables an interpretive synthesis that integrates empirical evidence, theoretical frameworks, and policy perspectives to develop a systemic understanding of a complex and multidimensional phenomenon. It is particularly suited to the study of complex systems and heterogeneous bodies of evidence, where the aim is to identify underlying mechanisms and recurring patterns across studies rather than to aggregate directly comparable empirical findings. By drawing on insights from multiple disciplines and methodological traditions, it extends beyond the more standardized scope typically associated with systematic or scoping review approaches.

### 2.3 Target group and concept

For this review, young people with disabilities were defined as individuals aged 0–25 years who experienced long-term and significant limitations in daily functioning and social participation due to chronic or developmental conditions.

In practice, this population includes subgroups commonly associated with elevated support needs and an increased risk of unmet mental health care, including intellectual and developmental disabilities, autism spectrum disorders, motor disorders, sensory impairments (including visual and hearing impairments), chronic illnesses, and developmental speech or language disorders. This typology provides a structured lens through which to organize and interpret a heterogeneous body of literature. It also reflects the reality that many studies and services remain organized around diagnostic categories even as broader functional and participatory perspectives are increasingly advocated.

In this review, specific learning disabilities (e.g., dyslexia and dyscalculia) and attention-deficit/hyperactivity disorder (ADHD) without co-occurring conditions were not included. While ADHD and specific learning disabilities are often discussed within neurodevelopmental frameworks and may involve significant psychological and educational challenges, the present review focused on disability contexts typically associated with enduring functional limitations and participation restrictions across multiple life domains, in line with a functional understanding of disability such as that proposed in the International Classification of Functioning, Disability and Health (15). In addition, the literature on ADHD and specific learning disabilities is extensive and largely organized around distinct research traditions. Including these conditions would have substantially expanded the scope of the review and risked obscuring the cross-cutting systemic mechanisms affecting populations with more pervasive support needs.

The inclusion of young people up to the age of 25 acknowledges the importance of transitions to adulthood, particularly the discontinuities encountered when moving from pediatric to adult mental health and disability care systems.

This review is grounded in a specific conceptual understanding of mental health, particularly in addressing the experiences of children and young people with disabilities. Mental health, as defined by the World Health Organization (27), is not simply the absence of mental disorders but a dynamic state of well-being in which individuals can realize their abilities, cope with the normal stresses of life, work productively, and contribute to their communities.

In line with the dual continua model (28), mental health and mental illness are understood as related but distinct dimensions rather than opposite ends of a single spectrum. Individuals may experience mental health problems while maintaining aspects of well-being and conversely may be free of diagnosable disorders yet not flourish.

This broader conceptualization underscores that mental health is shaped by complex interactions among biological, psychological, social, and environmental determinants. In disability contexts, these determinants include the accessibility and inclusiveness of physical and social environments, the quality of interpersonal relationships, the availability of adapted support, and opportunities for meaningful participation.

While this review adopts a broad understanding of mental health, the literature synthesized primarily addresses mental health problems and the organization of services responding to psychological distress and psychiatric comorbidities among young people with disabilities. These are considered across a continuum ranging from subclinical emotional and behavioral difficulties to formally diagnosed psychiatric disorders. The review also distinguishes between disability-related characteristics and co-occurring emotional or psychiatric difficulties, in line with work emphasizing the need to separate neurodevelopmental impairments from mental illness and mental health problems (29, 30).

Finally, while recognizing that disabilities generate specific support needs, this review stresses the diversity of identities and trajectories among young people with disabilities. Not all individuals primarily identify with a disability label; for some, their experiences are framed through cultural or community identities. Therefore, addressing mental health in this population requires approaches that are both flexible and culturally responsive, avoiding uniform models and grounding support for the lived meanings and priorities of young people and their families (31).

### 2.4 Procedure

#### 2.4.1 Literature sources and selection strategy

The literature analyzed in this review was collected as part of a broader interdisciplinary working group on inclusive mental health care established and conducted in Belgium. A structured search of peer-reviewed publications in English, French, and Dutch was conducted in PubMed, ScienceDirect, and Scopus in March 2024. Eligible publications included quantitative, qualitative, and mixed-methods studies, as well as systematic reviews and meta-analyses.

Given this review’s narrative and exploratory nature, a set of conceptual clusters guided the search process, combining terms related to mental health (such as “mental health,” “psychiatr*,” “prevalence,” “needs,” “support,” “care,” “treatment,” “guidelines,” “policy”…), target populations (such as “intellectual disabilit*,” “developmental disabilit*,” “autism spectrum disorder”…), age groups (such as “child*,” “adolescent*,” “young people,” “transition age”) and service organisation (such as “service delivery model”, “intervention”, “care system”…).

Searches relied primarily on keyword combinations applied to titles, abstracts, and indexed fields in each database. Database-specific search strings were adapted for each platform, and publication date filters were applied to include studies published between 1999 and 2024. The full search strategies used for each database are provided in Supplementary material.

The term *learning disabilities* was retained in the search strategy because, in UK usage, it is often used to refer to intellectual disabilities. During the screening process, particular attention was paid to exclude studies focusing exclusively on specific learning disorders (e.g., dyslexia, dyscalculia, dyspraxia) when these were not associated with broader developmental or intellectual disabilities.

Search results were imported into the Rayyan platform, which facilitated the collaborative screening process. Titles and abstracts were screened by one author, while two additional researchers each independently reviewed approximately 50% of the records. When reviewers identified uncertainties or potential disagreements regarding inclusion, these records were flagged for discussion. Decisions were then revisited collectively considering the review objectives and the predefined inclusion and exclusion criteria until consensus was reached. Given the narrative nature of the review, formal inter-reviewer agreement statistics were not calculated.

**Figure 1:**
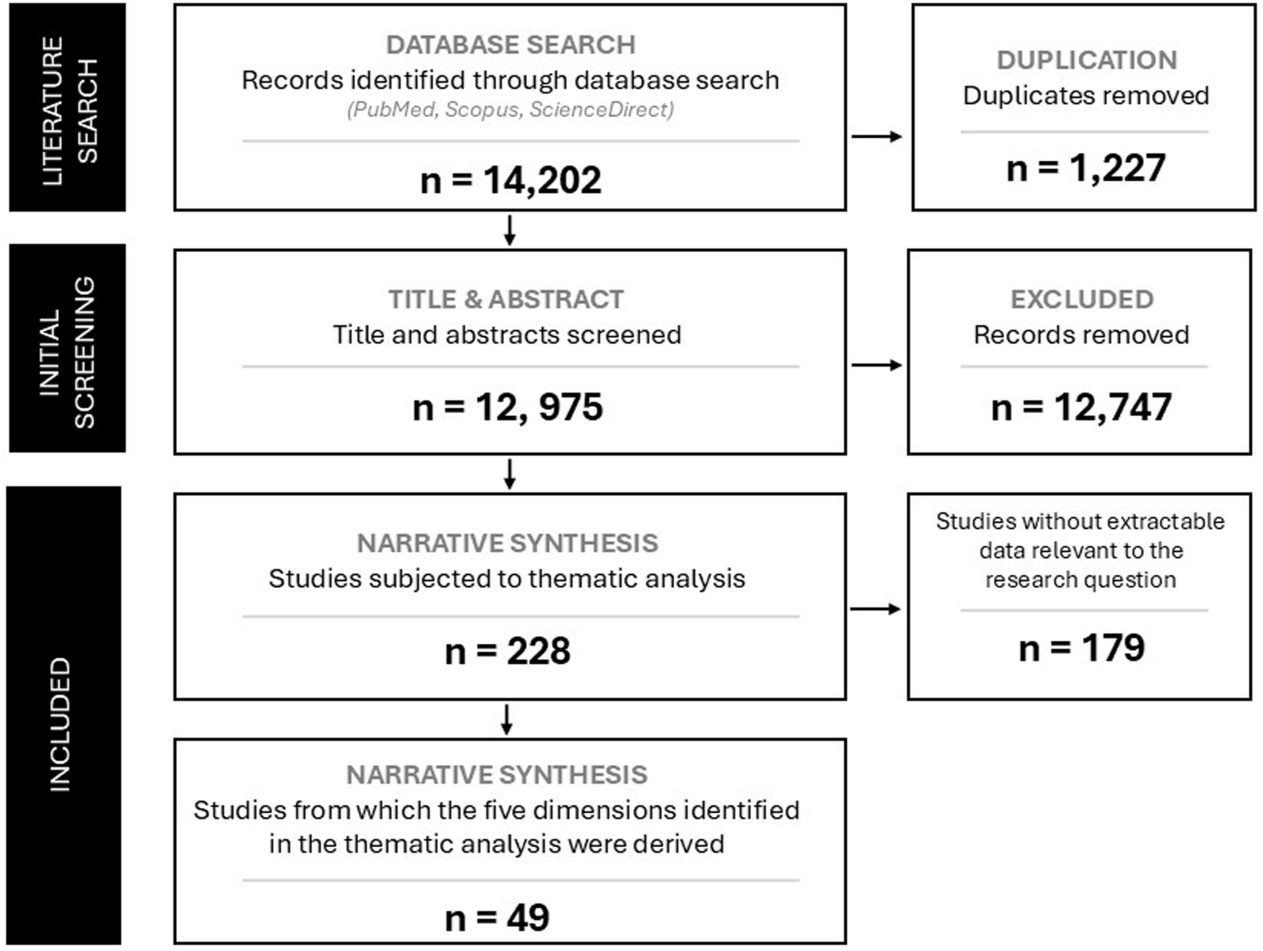
Flow diagram of the literature selection process.

The study selection process is illustrated in Figure 1. The database search identified 14,202 records, of which 1,227 duplicates were removed. A total of 12,975 records were screened at title and abstract level. Studies that clearly addressed the research question were retained for inclusion, resulting in a final corpus of 228 studies published between 1999 and 2024, which were subsequently subjected to thematic analysis.

Study selection followed a targeted and purposive approach aligned with the analytical aims of the review. Inclusion criteria were defined in relation to the population of interest (young people with disabilities), the focus of the study, and the type of issue addressed in relation to mental health. Studies were retained if they clearly and directly addressed at least one of the following: (1) the mental health or quality of life of young people with disabilities, including mental health problems across a continuum of severity from emotional and behavioral difficulties to formally diagnosed psychiatric disorders; (2) the experiences and mental health of their families (parents or siblings); or (3) the organization, accessibility, and responsiveness of mental health services for these populations. We excluded studies that focused exclusively on adult populations, narrowly defined interventions (such as a specific drug or therapy), isolated symptoms (such as sleep), or culturally and geographically specific contexts unlikely to inform service development in broader settings.

Additionally, gray literature was consulted, including policy reports and institutional documents, and the reference lists of selected articles were examined to identify complementary sources. Key international and national guidelines, including the United Nations Convention on the Rights of Persons with Disabilities, were also reviewed to inform the interpretation of service models and recommendations.

#### 2.4.2 Analytical process

The selected sources were subjected to qualitative thematic analysis, combining inductive coding with theoretical sensitization to concepts from disability studies, child development, and health equity. Coding was conducted using Atlas.ti. The analysis aimed to identify recurring themes related to systemic determinants shaping access to mental health care across disability contexts. Through iterative comparison across studies, codes were progressively grouped into broader conceptual categories, which ultimately structured the five cross-cutting dimensions presented in the results. Differences in interpretation and categorization were discussed and resolved collectively by the interdisciplinary research team. This collaborative process ensured cross-validation of perspectives from the clinical, educational, and social policy domains.

Because the aim of the review was to identify cross-cutting systemic determinants rather than to synthesize comparable empirical outcomes, a formal methodological quality appraisal of individual studies was not conducted. The review sought to extract information relating to service organization, access barriers, and participation across a highly heterogeneous body of literature addressing different primary research questions.

Finally, the literature reviewed encompasses a heterogeneous range of disability contexts. This diversity was explicitly acknowledged during analysis while maintaining a transdiagnostic perspective. Thematic coding therefore focused on identifying systemic determinants that recur across conditions, while noting when findings were reported in relation to specific diagnostic groups. Accordingly, the results are presented primarily at this cross-cutting level, except where the literature clearly relates to a particular condition.

## 3 Characteristics of sources of evidence

A total of 228 studies were screened and read as part of this narrative review. Among these, 49 studies directly informed the thematic analysis; a detailed overview of these studies is provided in Supplementary material 2. The broader corpus involved young people with chronic illnesses (*n* = 67, 29.4%), intellectual disabilities (*n* = 44, 19.3%), developmental speech or language disorders (*n* = 32, 14.4%), autism spectrum disorder (*n* = 27, 11.8%), hearing impairments (*n* = 23, 10.1%), motor disorders (*n* = 19, 8.3%), visual impairments (*n* = 11, 4.8%), or profound intellectual and multiple disabilities (*n* = 5, 2.2%).

## 4 Main dimensions emerging from the review

Based on the thematic analysis of the reviewed literature, five main dimensions were identified as particularly relevant to understanding the mental health of young people with disabilities and challenges related to care provision. These dimensions reflect the recurring themes, structural issues, and cross-cutting factors observed across diverse studies and sources. Each of these dimensions is discussed in detail in the following sections.

### 4.1 Service organisation and coordination

This dimension examines how the structural organization of mental health services and the level of coordination across sectors affect access to care for young people with disabilities. It considers the fragmentation of systems, lack of role clarity, and limited service integration, which frequently undermine the coherence and responsiveness of care trajectories in this population. These barriers are particularly significant given the complex, intersecting needs of many young people with disabilities, which require flexible, multidisciplinary, and coordinated approaches.

Across the reviewed literature, service provision for young people with disabilities was frequently described as fragmented and poorly integrated, undermining access to and continuity of care. Reports highlight the lack of systematic collaboration across mental health, education, social care, and disability-specific support, which often forces families to bridge the gaps between disconnected providers on their own. Families report a substantial administrative and navigational burden, with multiple points of contact and unclear roles that contribute to delays and frustration (5, 18, 20, 32–41). While a few local teams have attempted to mitigate these gaps through multidisciplinary clinics or embedding mental health expertise within rehabilitation- for example through coordinated cerebral palsy clinics bringing together neurology, neuropsychiatry and rehabilitation- the literature has consistently shown that these initiatives remain difficult to sustain and scale because of workforce shortages, organizational fragmentation and the significant resources required to support multidisciplinary care (32, 37, 42).

Beyond interagency issues, the literature notes intra-service inconsistencies. Within a single organization, professionals may operate under different mandates or frameworks with variable preparedness to address neurodevelopmental profiles or complex behavioral presentations. Several sources emphasize the need for multidisciplinary perspectives in assessments and interventions to address internal gaps. In particular, research stresses the importance of interdisciplinary or multidisciplinary approaches across conditions such as cerebral palsy (19, 37, 42, 43), hearing impairments (44), intellectual disabilities (45), autism and learning disorders (38, 46–50), as well as speech impairments (11, 51). These multidisciplinary perspectives are considered essential to ensure accurate diagnosis and targeted intervention and to improve care coordination, reduce service silos, and enhance the overall quality of life.

A recurrent point of discontinuity concerns transitions, particularly the shift from pediatric to adult services. Studies have described abrupt changes in eligibility, care philosophy, and professional expectations, often resulting in interruptions or even the loss of follow-up. These challenges have been consistently reported across disability groups and can significantly limit social participation (52). Evidence highlights the need for a lifespan perspective as developmental conditions persist across the life course and require long-term support strategies (47, 49). Families frequently describe the transition to adulthood as a time of uncertainty, given the loss of support previously available in childhood or through the education system (10, 35). Research also documents that outcomes in adulthood remain poor when appropriate preparatory interventions are lacking and when health and rehabilitation services are not maintained in young adulthood (2, 5, 53). Similar discontinuities have been widely documented in the broader transition-of-care literature in youth mental health systems, where the divide between child and adult services has long been recognized as a structural barrier to continuity of care (54–56). For young people with disabilities, these discontinuities may be particularly consequential given their strong reliance on coordinated service environments to support participation, functioning, and mental health across the life course. Overall, the literature underscores the need for sustained, developmentally attuned, and well-coordinated services across the transition period to avoid service gaps and ensure continuity of care.

### 4.2 Accessibility and availability of services

This dimension highlights the conditions in which mental health services are accessible to young people with disabilities. It refers to organizational aspects, and to families’ ability to identify, reach, and use services in a timely and effective manner.

Difficulties accessing information and navigating services were frequently described as barriers. Families often report difficulties in identifying relevant resources and navigating complex procedures. In some cases, professionals themselves lack awareness of specialized pathways, leading to delayed or inappropriate referrals. Studies have noted that limited access to appropriate psychosocial services can lead to an overreliance on pharmacological interventions, particularly when other forms of support are unavailable (35). Many individuals with developmental disabilities experience high rates of emergency and inpatient service use, reflecting the insufficient availability of outpatient care tailored to their needs (45). Families frequently report unmet needs, including respite, mental health care, and clear information about the available support (18). Across conditions, barriers such as a lack of coordinated care and difficulties obtaining referrals are repeatedly reported, leaving families with ongoing psychosocial and informational gaps (5, 36).

Eligibility criteria also emerged as restrictive factors. Access to services is frequently limited by narrow diagnostic labels or administrative thresholds that exclude children with complex or atypical presentations. In some contexts, children with learning disabilities are explicitly excluded from psychiatric services, leaving pediatricians to manage highly complex behavioral issues without adequate support (46). Families frequently report frustration when referrals to child and adolescent mental health services are rejected, and many parents feel that no suitable services are available for their children (11). Research also documents that individuals with intellectual disabilities are often redirected toward emergency or inpatient care because outpatient services lack the expertise to meet their needs (45). Simultaneously, children and adolescents who do not meet formal diagnostic thresholds may find themselves without access to support, which further contributes to cycles of unmet needs and discouragement for families (2).

Geographical disparities exacerbate inequality. Studies have highlighted the lack of specialist services in rural or underserved regions, forcing families to travel long distances at considerable financial and emotional costs (57, 58). Local services are often limited to general provisions without expertise in neurodevelopmental or communicative differences, which undermines the adequacy of the response. For example, access to psychotherapy for deaf people is often constrained by geographical location, leaving local counselors relatively unsupported and highlighting the need for a wider availability of psychotherapeutic interventions (59). Similarly, research on intellectual disabilities shows that despite the recognition of mental health needs, there are substantial regional variations in the availability of specialized clinical care, resulting in persistent underdiagnosis and inequitable access (33). Families from vulnerable groups, including those in rural or low-income areas, are less likely to access adequate health services, reinforcing systemic disparities (36). Practical barriers such as transport difficulties further limit the capacity of young people with disabilities and their families to access consistent care (5).

Financial barriers have consistently been reported. Even when mental health services are publicly covered, families face substantial out-of-pocket costs for assessment, therapy, and sustained intervention. Research highlights that inadequate or inconsistent insurance coverage is a recurring problem, with families reporting unmet needs, rejected referrals, and difficulties maintaining access to therapies (36). These barriers are particularly pronounced for children with developmental delays and intellectual disabilities, where gaps in insurance coverage further reduce the continuity of care (36). Families also describe financial constraints and insufficient funding as major obstacles to accessing necessary mental health services, contributing to frustration and discouragement (2). Altogether, lower-income families are significantly less likely to access specialist mental health care for their children despite comparable levels of need (35, 36).

Finally, functional accessibility of mental health services remains insufficient. Barriers extend beyond the physical environment and encompass communicative, sensory, and cognitive demands that restrict meaningful engagement. Physical obstacles, such as ramps, doorways, and toilets, continue to undermine equality of access (48). At the same time, many care settings rely heavily on verbal interactions, abstract reasoning, and written materials, with few adjustments to reduce the linguistic or cognitive load. This leaves children with speech, language, or cognitive difficulties at risk of confusion and exclusion unless alternative modes of interaction are actively integrated (11).

Environmental conditions also play a major role. Poor lighting, disruptive acoustics, excessive background noise, and overcrowded clinical spaces can exacerbate anxiety and overstimulation, especially in children with differences in sensory processing (44, 48). Similarly, clinicians’ reliance on simultaneous speech and action may create unnecessary cognitive overload in deaf children, who require clear sequencing to process information effectively (44). The absence of qualified interpreters or adapted communication strategies further compounds this inaccessibility, leaving children and families mistrustful or excluded from care (44).

Altogether, the literature underscores that functional accessibility is a matter of removing physical barriers and requires proactive attention to communication modes, emotional attunement, cognitive load, and sensory environments. Without these adjustments, therapeutic alliances and equitable participation in mental health care remain compromised.

### 4.3 Adequacy of services

This dimension examines the extent to which the existing mental health services respond to the diverse and often complex needs of young people with disabilities. Beyond access, the question arises of whether available care is sufficiently adapted, individualized, and responsive.

Limitations of generalist frameworks have been widely noted. Many services rely on standardized models that fail to capture the multifaceted profiles of youths with disabilities, leading to insufficiently individualized interventions and persistent unmet needs. Clinicians without experience in disability-specific contexts often misinterpret signs and apply inadequate treatment approaches, partly because of poor communication skills with certain populations, limited knowledge of developmental pathways, and a lack of validated tools (60). This challenge is compounded when comorbid psychiatric, behavioral, or somatic conditions are present, as the recognition and management of such complexity remain areas of weakness in training and practice (61, 62).

Service environments also reflect this misalignment: Rehabilitation settings may prioritize physical health but lack the awareness and skills to identify or address co-occurring mental health needs (5), while outpatient clinics often report limited staff, insufficient training, and inadequate knowledge of resources to support children with complex profiles (63). Together, these findings suggest that reliance on generalist frameworks obscures the multidimensionality of young people’s needs and perpetuates systemic barriers to timely and appropriate care.

A recurring problem is diagnostic overshadowing (64), whereby emotional or behavioral difficulties are attributed to the disability itself rather than being recognized as distinct psychiatric conditions. This misattribution contributes to the under-recognition of mental health needs and delays access to appropriate interventions. For example, developmental differences in deaf youths may be misinterpreted as signs of unrelated psychiatric disorders such as ADHD or psychosis, leading to inappropriate diagnoses (44). Similarly, the overlapping features of deafness and autism can obscure the diagnostic picture and lead to overshadowing, resulting in delays in treatment (60). In intellectual disabilities, clinicians often classify symptoms such as sadness or irritability as inherent to the disability rather than as markers of co-occurring mental health problems (33, 65, 66). Moreover, somatic comorbidities are frequently overlooked, with evidence showing that untreated medical issues drive a significant proportion of psychiatric admissions among young people with intellectual disabilities (45). Together, these findings highlight how overshadowing distorts the recognition of psychiatric needs, reinforces diagnostic inequities, and contributes to prolonged unmet needs. Beyond the diagnostic processes, families experience overshadowing as a relational barrier. Parents reported that inadequate professional knowledge of communication needs can undermine therapeutic alliances and lead to children being perceived as uncooperative (11). Among deaf youths, families report feelings of fear, mistrust, and frustration in health care settings, particularly when communication is mishandled -for instance, when side conversations with interpreters are perceived as exclusionary (44). Disability-associated stigma further discourages help-seeking and disclosure, amplifying the sense of dismissal and judgment encountered by families (5, 20). Together, these findings highlight how diagnostic overshadowing distorts the clinical recognition of psychiatric needs and fosters negative professional–family dynamics, weakening trust and engagement in care.

The lack of professional training has compounded these issues. Practitioners frequently report feeling unprepared to work with children with intellectual and developmental disabilities, particularly when confronted with complex behavioral or psychiatric presentations. Pediatric providers themselves recognize that enhanced education and exposure could reduce the high levels of unmet health needs among children with ID and ASD (36). Yet across systems, there are insufficient numbers of adequately trained specialists (18). Clinicians working with young people with physical disabilities describe feeling unequipped to manage mental health needs as rehabilitation-focused training leaves them with limited awareness of psychiatric issues (5, 20). Similar concerns have emerged in somatic outpatient clinics, where a lack of staff training and knowledge of local resources exacerbates barriers to care (63).

Training gaps also manifest in psychiatry, where the recognition and management of comorbid emotional, behavioral, and language disorders remain an area of weakness despite decades of evidence of their prevalence (62). Parents further report that inadequate professional knowledge of language and communication needs can undermine therapeutic alliances and leave children feeling misunderstood or labeled uncooperative (11). Collectively, these findings highlight the urgent need for specialized, neurodevelopmentally informed training across all tiers of service provision to replace the reliance on generic protocols with flexible, individualized approaches. These gaps are not solely due to a lack of training opportunities but also reflect deeper structural and cultural issues within the medical field. Psychiatry and medicine have historically demonstrated limited engagement across many disability contexts. These populations are often perceived as complex, slow to respond to therapy, and less attractive from career or research perspectives. Consequently, they remain underrepresented in training curricula and clinical practice, which perpetuates the scarcity of specialized expertise and limits the development of tailored and disability-informed mental health care.

Finally, there are critical gaps in tools and interventions. There are few validated assessment instruments for atypical or complex profiles, and limited research has been dedicated to their development, which restricts both diagnostic accuracy and timely support. For example, while many measures are available for challenging behaviors, no standardized instruments exist to assess trauma in people with developmental disabilities, and validated tools to capture emotions remain scarce (67, 68). In children and young people with hearing impairments, the lack of standardized and validated tools increases the risk of misinterpretation and misdiagnosis (60, 69). Similar concerns have been echoed in the field of speech impairment, where a shortage of instruments has been noted, particularly for stuttering in school-aged children (70).

Intervention frameworks rely heavily on verbal and abstract cognitive tasks that exclude children with speech, language, or cognitive impairments. Parents reported that a lack of adaptive strategies can threaten therapeutic alliances, whereas approaches that reduce linguistic demands and integrate alternative activities are more effective (11). Further evidence suggests that personalized and modified psychological interventions, when properly tailored, can significantly improve outcomes and continuity of care (43). Collectively, these findings highlight the urgent need to develop and disseminate evidence-based tools and interventions that reflect the diversity of developmental profiles.

### 4.4 Participation of young people and families

This dimension examines the extent to which young people with disabilities and their families are actively involved in shaping mental health care they receive. The literature consistently stresses that they should not be treated as passive recipients of support but as partners whose knowledge and perspectives are central to effective care. However, across studies, participation is limited and constrained by assumptions about competence, unequal power dynamics, and a lack of accessible communication.

At the individual level, meaningful participation is often compromised. Young people are rarely supported in expressing their views or being directly involved in decisions regarding their care. Instead, adults-most often parents-are treated as the sole interlocutors. Communication barriers, inaccessible information, and professional assumptions regarding competence reinforce this dynamic. Evidence shows that reliance on proxy reports can distort the understanding of children’s well-being, underlining the importance of incorporating self-reports whenever possible (35, 71). Approaches that emphasize child-reported outcomes and prioritize children’s own perspectives are more consistent with person-centered care (47, 50). In practice, this means engaging children directly in their preferred mode of communication, integrating alternative methods, such as visual tools or play-based strategies, to support expression, and tailoring interventions around individual needs (11). Collaborative models that position young people as active partners rather than passive recipients also strengthen participation and improve quality-of-life outcomes (52).

At the organizational level, parents and caregivers reported few genuine opportunities to influence service design or evaluation. While occasional consultations or surveys exist, families often describe them as tokenistic exercises -gestures of inclusion with no real impact on decision-making. Consequently, services remain insufficiently responsive to families’ lived realities (10, 11, 72). In contrast, the literature emphasizes that meaningful shared decision-making requires structured mechanisms, such as advisory boards, co-design processes, and ongoing collaboration between professionals and families (52, 73).

Crucially, this collaboration depends on recognizing parents as experts on their children’s history, preferences, and communication styles. Families consistently highlight the value of their experiential knowledge but also report that this expertise is frequently undervalued. When parents feel dismissed or sidelined, trust in services weakens and engagement falters. Empowerment, therefore, requires formal participation structures and practical tools and resources that allow parents to act as confident partners in care. Strategies such as guidance on understanding developmental or language-related difficulties, structured routines, and visual support help families contribute effectively to decision-making and daily management (51, 62). Moving beyond symbolic consultation toward genuine partnerships strengthens service relevance, enhances trust, and lays the foundation for sustainable family-centered care.

Finally, several sources have highlighted the psychological strain of caregiving. Parents frequently report feelings of stress, isolation, and fatigue linked to ongoing demands to support a child with disabilities. Large proportions explicitly describe the need for emotional support, respite opportunities, child-focused mental health care, and clear information (18). Without such resources, many families report feeling overwhelmed and helpless, especially when confronted with severe mental health difficulties. In these situations, access to psychological support for parents becomes essential, both for their own well-being and to sustain their capacity for care (20). Families also emphasize the need for guidance and tools to manage behavioral and emotional challenges in daily life (51). In some groups, structural barriers further exacerbate vulnerability; for instance, deaf mothers deprived of adequate support and preparation for parenting report a lack of coping skills and knowledge (59). Taken together, these findings underscore that supporting parents is not optional; it is a prerequisite for both family well-being and children’s access to effective and sustained mental health care.

### 4.5 Stigma

Social and institutional stigma has emerged as consistent barriers to mental health care. Anticipated stigma deters many families from seeking support because concerns about being judged or misunderstood contribute to delays and non-disclosure (5, 20). Professional stigma compounds these difficulties; deficit-based assumptions often result in diagnostic overshadowing, where psychiatric symptoms are attributed to disability rather than being recognized as co-occurring conditions (33, 44, 60). Families also report interactions in which their concerns are dismissed or reframed as behavioral non-cooperation, weakening therapeutic alliances (11). At a structural level, institutional norms around communication and competence, such as reliance on relatives as interpreters, reinforce exclusion and create environments where families feel “out of place” (59). Collectively, these dynamics constrain access, reduce engagement, and perpetuate care inequality.

## 5 Discussion

This review sought to understand how structural, organizational, and relational factors interact to shape access to and the quality of mental health care for young people with disabilities. Building on this aim, the following discussion interprets the findings in light of these objectives and considers their implications for the development of more inclusive and coordinated mental health systems. Before discussing the implications of our findings, it is important to highlight the methodological distinctiveness of this review. Extracting insights into the organization and coordination of mental health services for young people with disabilities requires a line-by-line analysis of studies that are rarely designed to address system-level questions. Despite including service organization and coordination as explicit selection criteria, very few of the retained studies explicitly addressed these issues as their primary focus. Instead, the literature is largely structured around single diagnostic categories, with most studies examining narrow therapeutic options, specific parental variables, or isolated behavioral or cognitive components. This fragmented landscape reflects the siloed nature of the evidence base and also underscores the lack of integrated perspectives that span conditions and settings. By systematically tracing and collating scattered mentions of service-related issues across diverse studies, our review helps fill this gap. It highlights the urgent need to move toward more comprehensive transdiagnostic approaches to service design and evaluation. Such approaches should view people with disabilities through the lens of their needs and potential, rather than their limitations, thereby promoting more inclusive, equitable, and diverse service offerings tailored to individual circumstances.

Briefly, our results converged in five main areas. First, service provision is characterized by fragmentation and inconsistent coordination, leaving families to bridge gaps between disconnected providers. Second, access is constrained by barriers to navigation, eligibility, geographical disparities, financial constraints, and functional access. Third, the adequacy of services is undermined by reliance on generalist frameworks, diagnostic overshadowing, training gaps, and scarcity of adapted tools and interventions. Fourth, participation by both young people and families is limited, with decision-making still largely adult-driven and with few opportunities for co-design or sustained empowerment. Finally, anticipated, professional, and structural forms of stigma emerge as cross-cutting barriers. These dimensions span several levels of the mental health system, ranging from structural conditions shaping coordination and accessibility to relational and experiential aspects such as participation and stigma. In this sense, they align with broader conceptualizations of health care access and health systems that emphasize the interaction between service organization and users’ experiences (74, 75).

Taken together, the findings suggest that some determinants appear more consistently across disability groups than others. Across a wide range of contexts, including intellectual disabilities, autism spectrum conditions, motor impairments, hearing impairments and language disorders, the literature repeatedly points to structural weaknesses in service systems. Fragmentation between sectors, limited coordination of care, insufficient availability of specialized mental health services and gaps in professional training emerge as recurring systemic barriers affecting access to mental health support.

By contrast, other determinants appeared across multiple disability groups but were expressed in more context-specific ways, shaped by the functional characteristics of each condition. Communication barriers, for instance, were consistently reported but required fundamentally different adjustments depending on the population: sign language interpretation and visual communication supports for deaf children, simplified language or augmentative and alternative communication strategies for children with intellectual or language impairments, and modifications to sensory environments for children with sensory processing differences. Similarly, accessibility barriers and issues of diagnostic overshadowing were pervasive across groups, but their concrete manifestations - and therefore the adaptations required - differed considerably.

This distinction has practical implications. It suggests that the most persistent inequities relate to the structural organization and preparedness of service systems, and that these should be prioritized in cross-cutting policy and training reforms. At the same time, the modalities of care must remain adapted to the specific functional and communicative characteristics of different disability groups, resisting any tendency toward uniform solutions.

The convergence of findings across dimensions suggests that systemic barriers are unlikely to be meaningfully addressed through isolated adjustments alone. Instead, they call for adopting a universal design for healthcare grounded in strategic changes to how services are coordinated, made accessible, adapted to diverse needs, and shaped together with young people and families. To move from fragmented evidence to actionable directions, we outline a series of recommendations structured around the five dimensions identified in our review.

The reviewed evidence points toward the need to move beyond fragmented and crisis-driven provisions, suggesting that more coherent, sustained, and inclusive responses may better address the complexity of mental health needs in this population. The literature emphasizes the importance of stronger coordination mechanisms across health, education, and social care, as the lack of integration often leaves families acting as default coordinators, navigating multiple points of contact with unclear responsibilities (18, 33). To alleviate this burden, services should establish clear referral pathways and single points of contact to ensure that families do not carry the primary responsibility for linking professionals. Particular attention is needed at transition points, especially during the shift from pediatric to adult services. The evidence highlights abrupt discontinuities, leaving young people without sustained support (2, 5, 10, 35). This underlines the importance of anticipatory planning for transitions, co-construction with family, and extending follow-up to early adulthood.

Accessibility is another critical aspect of action. Families consistently report unmet respite needs, child-focused mental health care, and clear information about available resources (18, 76). Navigation support and accessible materials adapted to different linguistic and cognitive profiles emerge as particularly important levers for reducing barriers to access. Regional disparities also require targeted strategies; in many contexts, specialist services are concentrated in urban centers, obliging families in rural areas to travel long distances (36, 77). Mobile specialist teams or hybrid models combining in-person and remote consultations can reduce these inequities. At the same time, eligibility criteria remain a structural barrier, with children excluded because their difficulties do not fit narrow diagnostic categories or because they are deemed “too complex” for standard provision (33, 78, 79). Broadening the access criteria to focus on functional needs rather than diagnostic labels would reduce the number of cycles of exclusion and crisis-driven referrals.

Even if families succeeded in accessing these services, their adequacy remained a concern. Studies emphasize the risk of misdiagnosis or inappropriate interventions when assessments are conducted by a single professional or based on standardized models ill-suited to complex profiles (60, 69). To address this, services should embed multidisciplinary and multi-informant assessment practices and systematically integrate perspectives from parents, teachers, and specialists (44, 45, 71). This reduces the risk of diagnostic overshadowing and enables more targeted interventions. The lack of adapted diagnostic and therapeutic tools has also emerged as a recurring issue. While existing measures rely heavily on verbal or abstract reasoning, they exclude children with communication or cognitive impairments (67, 68, 70). Immediate improvements could be achieved by adapting existing tools, such as simplified language, visual support, or play-based methods. At the same time, long-term efforts should focus on developing validated instruments tailored to diverse developmental profiles. Professional training is a prerequisite for this change. Clinicians frequently report feeling ill-equipped to address mental health needs in young people with disabilities (20, 36, 65), underscoring the need for embedding disability- and neurodiversity-informed training in both initial education and continuing professional development (78, 79).

The participation of young people and their families should be strengthened at every stage of care. Children’s perspectives are often bypassed either because of assumptions about competence or because services rely exclusively on verbal communication. Evidence shows that self-reporting, when supported by adapted methods, provides unique insights into children’s experiences (35, 71, 80). These findings highlight the value of integrating alternative communication strategies- such as visual tools, pictograms, role-play, or other accessible methods- to better capture children’s views directly (11, 44, 81). Families, for their part, provide indispensable knowledge of their child’s history, preferences, and communication style, yet they often feel sidelined or judged by professionals. Recognizing parents as partners and supporting them with tools such as structured routines, visual aids, and guidance on managing behavioral challenges would enable a more balanced collaboration (20, 62). Involving experienced parents as peer mentors within service teams can also enhance family empowerment and trust in services (20).

Support for family and caregivers emerges as a central component of effective care. Parents describe the cumulative stress, fatigue, and isolation associated with caregiving, highlighting the need for accessible respite, psychological support, and structured preparation for parenting in the context of disabilities (18, 82, 83). Ensuring that caregivers’ needs are systematically assessed and supported is not an optional addition to child-focused services, but a necessary condition for sustaining care (20, 84). Without adequate recognition of parental well-being, both family stability and children’s access to mental health care are compromised (2, 5).

Stigma emerged across several studies as a factor that may operate at multiple levels, warranting attention alongside more structural determinants of access. Anticipated stigma deters young people and families from seeking help (5, 20), whereas professional stigma-diagnostic overshadowing, parental blame, and assumptions of incompetence-undermines therapeutic alliances (10, 85, 86). Structural stigma persists in institutional cultures that rely on inaccessible communication norms or that position families as outsiders (86). Tackling stigma is not peripheral; it is a precondition for equitable access, sustained engagement, and trust in mental health care.

Taken together, these recommendations suggest that improving mental health care for young people with disabilities likely requires attention to multiple, interconnected levels rather than isolated adjustments alone. What emerges across conditions is the need for structures that are coordinated, inclusive, flexible, and grounded in the recognition of both children’s and families’ expertise. While most of the included studies were not designed to address service systems directly, analysis of their findings highlighted common entry points for change: clear coordination mechanisms, adapted assessment and intervention tools, structured participation opportunities, and systematic support for caregivers. Addressing stigma and professional culture is equally crucial, as without these shifts, even well-designed models risk reproducing barriers. Ultimately, a transdiagnostic and systemic approach is necessary to improve access and adequacy of care in the short term, and to build conditions for equitable, sustainable service provision across the life course.

This study had several limitations. As a narrative rather than systematic review, the literature selection was purposive and thematic, and relevant studies may have been overlooked. In addition, the evidence base was highly heterogeneous, encompassing diverse disability groups, age ranges, methodologies, and outcome measures, which limited direct comparability across studies. Many of the included studies relied on qualitative or descriptive designs, and robust experimental evidence remains scarce, particularly regarding service organization and participation outcomes. Another limitation concerns the geographical distribution of the literature, which largely originates from high-income countries and may therefore limit the transferability of findings to other health care and policy contexts. Moreover, by focusing on cross-cutting mechanisms and shared barriers, this review may not fully capture the condition-specific nuances that shape mental health trajectories. Furthermore, while the screening process involved partial independent verification and collective discussion of uncertainties, the primary screening was conducted by one reviewer, which is consistent with narrative review practices but may have introduced some selection variability. Finally, as the objective was to identify recurring systemic mechanisms across a heterogeneous body of literature, a formal methodological quality appraisal of individual studies was not conducted. The lack of a formal quality appraisal limits our ability to assess the strength of the evidence and to weigh findings according to their methodological rigor, potentially allowing lower-quality studies to influence the conclusions. Accordingly, the conclusions drawn from this synthesis should be interpreted with caution, particularly regarding the strength and generalizability of the identified patterns.

Despite these limitations, the synthesis offers a valuable overview of the structural and systemic dynamics that influence access to mental health care for young people with disabilities and identifies key areas for future research and policy development.

While previous reviews have typically focused on specific diagnostic groups, prevalence estimates, or service models, they rarely examine how systemic factors interact across disability contexts to shape access to mental health care. By adopting a transdiagnostic and systems-oriented perspective, the present synthesis integrates evidence across diverse disability groups and service settings to identify a set of cross-cutting structural and relational determinants influencing how mental health care becomes accessible, coordinated, and responsive for young people with disabilities.

In summary, the five dimensions identified in this review -coordination, accessibility, adequacy, participation, and stigma -offer a preliminary framework for rethinking service design and delivery. Future work should focus on translating these insights into operational guidance co-developed with young people, families, practitioners, and policymakers. Moving beyond diagnostic silos and deficit-oriented frameworks, toward inclusive and participatory systems, appears to be an important condition for mental health care to become more equitable and developmentally responsive for young people with disabilities.

More broadly, embedding a universal design framework within mental health care has the potential to reshape research, training, and policymaking. It calls for research agendas that prioritize cross-cutting determinants of inclusion and equity, training programs that equip professionals with competencies in accessibility, communication, and co-production, and policy structures that promote intersectoral coordination and shared accountability. Through such alignment, universal design becomes not merely a service principle but a foundation for building mental health systems conceived for diversity rather than adapted to exception.
