# Supplementary material for: Transdiagnostic determinants of access to mental health care for youth with disabilities: a systems-oriented structured narrative review
Source: Front Public Health. 2026 May 12;14:1782003. doi: 10.3389/fpubh.2026.1782003 (PMC13202938; doi:10.3389/fpubh.2026.1782003)
Supplement: Supplementary file 1 [file Data_Sheet_1.pdf]

Supplementary Material 1

Database search strategies

PubMed search strategy

("mental health"[Title/Abstract] OR "mental healthcare"[Title/Abstract] OR psychiatr*[Title/Abstract] OR "psychiatric disorder*"[Title/Abstract] OR "behavioral health"[Title/Abstract])
AND
(child*[Title/Abstract] OR adolescent*[Title/Abstract] OR "young people"[Title/Abstract] OR "young adult*"[Title/Abstract] OR "transition age"[Title/Abstract])
AND
(disability[Title/Abstract] OR disabilities[Title/Abstract] OR disabled[Title/Abstract] OR "autism spectrum disorder"[Title/Abstract] OR ASD[Title/Abstract] OR "intellectual disabilit*"[Title/Abstract] OR IDD[Title/Abstract] OR "learning disabilit*"[Title/Abstract] OR "developmental disabilit*"[Title/Abstract] OR deaf*[Title/Abstract] OR blind*[Title/Abstract] OR "visual impairment"[Title/Abstract] OR deafblind*[Title/Abstract] OR "chronic ill*"[Title/Abstract] OR "motor disorder"[Title/Abstract] OR polyhandicap*[Title/Abstract] OR "cerebral palsy*"[Title/Abstract])
AND
(organiz*[Title/Abstract] OR facilit*[Title/Abstract] OR administrat*[Title/Abstract] OR "service delivery model"[Title/Abstract] OR system*[Title/Abstract] OR care[Title/Abstract] OR coordination[Title/Abstract] OR continuity[Title/Abstract] OR "patient-centered"[Title/Abstract] OR treatment[Title/Abstract] OR psychotherap*[Title/Abstract] OR intervention[Title/Abstract] OR pharmacotherap*[Title/Abstract] OR "evidence-based"[Title/Abstract] OR prevent*[Title/Abstract] OR "early intervention"[Title/Abstract] OR "risk reduction"[Title/Abstract] OR "social support"[Title/Abstract] OR "peer support"[Title/Abstract] OR "family support"[Title/Abstract] OR communit*[Title/Abstract] OR need*[Title/Abstract] OR prevalence*[Title/Abstract] OR incidence[Title/Abstract] OR epidemiolog*[Title/Abstract] OR guideline*[Title/Abstract] OR polic*[Title/Abstract])
AND
("1999"[Date - Publication] : "2024"[Date - Publication])

Scopus search strategy

TITLE-ABS-KEY ("mental health*" OR "mental healthcare" OR "behavior* health*" OR psychiatr* OR "psychiatric disorder*")
AND TITLE-ABS-KEY (child* OR adolescent* OR "young people" OR "young adult*" OR "transition age")
AND TITLE-ABS-KEY (disability OR disabilities OR disabled OR "autism spectrum disorder" OR ASD OR "intellectual disabilit*" OR IDD OR "learning disabilit*" OR "developmental disabilit*" OR deaf* OR blind* OR "visual impairment" OR deafblind* OR "chronic ill*" OR "motor disorder" OR polyhandicap* OR "cerebral palsy*")

AND TITLE-ABS-KEY (organiz* OR facilit* OR administrat* OR "service delivery model" OR system* OR care OR coordination OR continuity OR "patient-centered" OR treatment OR psychotherap* OR intervention OR pharmacotherap* OR "evidence-based" OR prevent* OR "early intervention" OR "risk reduction" OR "social support" OR "peer support" OR "family support" OR communit* OR need* OR prevalence* OR incidence OR epidemiolog* OR guideline* OR polic*)

AND PUBYEAR BETWEEN 1999 AND 2024

ScienceDirect search strategy

("mental health*" OR "mental healthcare" OR "behavior* health*" OR psychiatr* OR "psychiatric disorder*")

AND (child* OR adolescent* OR "young people" OR "young adult*" OR "transition age")

AND (disability OR disabilities OR disabled OR "autism spectrum disorder" OR ASD OR "intellectual disabilit*" OR IDD OR "learning disabilit*" OR "developmental disabilit*" OR deaf* OR blind* OR "visual impairment" OR deafblind* OR "chronic ill*" OR "motor disorder" OR polyhandicap* OR "cerebral palsy*")

AND (organiz* OR facilit* OR administrat* OR "service delivery model" OR system* OR care OR coordination OR continuity OR "patient-centered" OR treatment OR psychotherap* OR intervention OR pharmacotherap* OR "evidence-based" OR prevent* OR "early intervention" OR "risk reduction" OR "social support" OR "peer support" OR "family support" OR communit* OR need* OR prevalence* OR incidence OR epidemiolog* OR guideline* OR polic*)

Filters applied in database interface for ScienceDirect: publication years 1999–2024.

Search date: March 2024

Supplementary Material 2

Table summarizing the studies from which the five dimensions identified in the thematic analysis were derived

References	Country	Design	Target population	Main objective	Key findings
**Allington-Smith et al., 2006**	UK	Opinion article	Children and adolescents with learning disabilities	Describe and raise public awareness of the mental health needs of children with learning disabilities, and advocate for improved specialized psychiatric care.	Children with learning disabilities have a high prevalence of mental health disorders, influenced by comorbidities, communication difficulties, and environmental factors, and require multidisciplinary care.
**Arthur, 2003**	UK	Narrative review	Children and adolescents with learning disabilities	Review existing research and clinical knowledge on the emotional lives and difficulties of people with learning disabilities.	People with learning disabilities show higher levels of emotional and developmental difficulties, requiring multidisciplinary care to improve quality of life.
**Augestad, 2017**	Norway	Systematic review	Children and young adults with visual impairment	Summarize current scientific knowledge relating to the occurrence of mood disorders among children with visual impairments	Social support, friendship, and independence in mobility seem to be important for enhancing the mental health of all children. Children with earlier onset and more severe visual impairments may be less likely to experience a reduction in their mental health problems over time.
**Bernard, 2009**	UK	Opinion article	Children and adolescents with learning disabilities	Explore the range, presentation assessment, and management of mental health and behavioural problems in children and adolescents with learning disability, compares and contrasts these children with those without learning disability.	Children and adolescents with learning disabilities have a higher risk of complex and persistent mental health and behavioural problems compared to their peers. These difficulties impact both the individual and their environment, requiring a range of coordinated services.

**Berney, 2006**	UK	Opinion article	Children and adolescents with learning disabilities	Provide an overview of learning disabilities in children, including their assessment, associated psychiatric disorders, and multidisciplinary management.	Effective care for children with learning disabilities requires multidisciplinary collaboration, with psychiatry as one component, emphasizing family support, prevention, and careful diagnosis to avoid mis- or overdiagnosis.
**Bigler et al., 2019**	USA	Systematic review	Deaf or hard-of-hearing children and young adults	Assess the association of behavioral disorders among children who are deaf and hard of hearing and to explore behavioral interventions for children in this population.	Although behavioural problems are well documented in deaf and hard of hearing children, the underlying mechanisms and effective interventions remain insufficiently understood. Further research is needed to develop targeted strategies and reduce long-term adverse outcomes.
**Bjorgaas et al., 2022**	Norway	Cohort Study	Pre-adolescents with cerebral palsy	Assess changes in parent-rated mental health problems in a cohort of children with CP from school-starting age to pre-adolescence, differences in mental health problems according to informants and the validity of the Strengths and Difficulties Questionnaire (SDQ) for psychiatric disorders.	Parents and pre-adolescents reported higher levels of emotional and behavioural problems than teachers, while children themselves reported a lower perceived impact. The SDQ was found to be a valid tool for screening psychiatric risk in children with cerebral palsy at pre-adolescence.
**Broad et al., 2017**	Canada	Qualitative design	Young people with learning and intellectual disabilities transitioning between mental health services	Provide a more comprehensive understanding of youth experiences of transition from Child and Adolescent Mental Health Services (CAMHS) to Adult Mental Health Services (AMHS), through a qualitative thematic synthesis of the extant literature in this area.	Youth experience a dramatic culture shift between CAMHS and AMHS, which can be mitigated by individualized and flexible approaches to transition. Youth have valuable perspectives to guide the intelligent design of mental health services, and their perspectives should be used to inform tools to evaluate and incorporate youth perspectives into transitional service improvement.

**Buckley et al., 2020**	Australia	Systematic review and meta-analysis	Children and adolescents with intellectual disability	Address inconsistencies in prevalence estimates of psychiatric symptoms and disorders in children and adolescents with intellectual disability, by examining the influence of measurement methods and subgroup characteristics such as severity, gender, and age.	Mental health comorbidities in individuals with intellectual disability may be better detected using symptom-based rather than diagnostic approaches. High prevalence rates emphasize the need for valid measurement tools, systematic screening, and appropriate management.
**Charlot et al., 2022**	USA	Cross-sectional study	Children and adolescents with intellectual disability	Determine the extent to which individuals seen in the clinic carried psychiatric diagnoses at the time of referral that were different from or the same as those identified by the multidisciplinary team after a comprehensive case evaluation process conducted by clinicians with experience assessing individuals with intellectual and developmental disabilities.	Individuals with intellectual disability, with or without ASD, have complex behavioural health needs that are often unresponsive to usual care. The Mood and Anxiety Semi-Structured interview tool (MASS) shows promise as an assessment tool, particularly for identifying anxiety and depression.
**Colver, 2016**	UK	Opinion article	Children and adolescents with cerebral palsy	Review children's outcomes in individuals with cerebral palsy, including life expectancy, quality of life, participation, and physical and mental health	Children with cerebral palsy have poorer outcomes than the general population across quality of life, participation, and health domains, although life expectancy is near normal without severe comorbidities.
**Curran et al., 2001**	UK	Cross-sectional study	Children with severe disabilities	Assess the time costs of caring for children with severe disabilities in the community compared to children without disabilities using diary- and questionnaire-based methods.	Children with severe disabilities require substantially higher and persistent levels of care, which significantly impacts family socioeconomic status, particularly limiting maternal employment. Improved assessment tools, such as a functional disability score, may support more equitable allocation of resources.

**Downs et al., 2018**	Australia	Systematic review and meta-analysis	Children and adolescents with cerebral palsy	Describe the prevalence of mental health conditions and problems in children and adolescents with cerebral palsy.	More studies are needed to ascertain the prevalence of mental health disorders. Mental health symptoms are common and mental health evaluations should be incorporated into multidisciplinary assessments for these children.
**Dreyzehner et al., 2019**	USA	Narrative review	Deaf or hard-of-hearing young adults	Review depression in deaf and hard-of-hearing youth, including its prevalence, risk factors, and considerations for assessment and management.	Multiple factors, including communication difficulties, family stress, and adverse experiences, increase the risk of depression in deaf and hard-of-hearing youth. Effective care requires minimizing language barriers and promoting psychosocial skills such as communication and emotion regulation.
**Drmic et al., 2017**	Canada	Narrative review	Children and adolescents with autism spectrum disorders	Apply a life course health development framework to autism spectrum disorders (ASD) in order to highlight key issues and guide research and interventions across the lifespan	Despite significant advances in ASD research, important gaps remain, particularly in optimizing life course outcomes. The life course health development framework offers a valuable approach to guide future research and improve outcomes for individuals with ASD and their families.
**Dykens, 2007**	USA	Narrative review	Children and adolescents with Intellectual disability (Down syndrome)	Summarize key findings on the type and prevalence of behavior and emotional problems in children, adolescents, and adults with Down syndrome.	Future research should focus on longitudinal understanding of psychiatric outcomes across development, as well as the complex biopsychosocial factors influencing mental health in individuals with Down syndrome. Additionally, there is a need to identify evidence-based interventions that reduce symptoms and improve overall well-being.

**Dykens, 2000**	USA	Narrative review	Children with Intellectual disability	Review recent advances in the understanding of psychopathology in children with intellectual disability, with a focus on prevalence, measurement issues, and underlying etiological factors.	Higher risk of psychopathology in children with intellectual disability, driven by complex bio-psycho-social factors, including genetic influences.
**Gagnon-Roy et al., 2016**	Canada	Scoping Review	Adolescents with motor impairment	Synthesize the current knowledge on social participation, which is the performance of an individual in realizing his or her daily activities and social roles within its life environment.	All areas of daily life are affected in adolescents and young adults with DCD, particularly education and interpersonal relationships, with new challenges such as driving emerging over time. Mental health difficulties are prominent, yet few effective strategies or interventions are described to support their social participation.
**Gentili and Holwell, 2011**	UK	Narrative review	Deaf or hard-of-hearing young adults	Examine how early language deprivation contributes to emotional and behavioural difficulties, emotional dysregulation, and the potential misdiagnosis of neurodevelopmental disorders in deaf children and adolescents.	Children with severe hearing impairment are at increased risk of psychiatric and psychosocial difficulties mainly due to language barriers, highlighting the importance of early diagnosis and effective communication support. Accurate assessment and management require specialized, multidisciplinary care to avoid misdiagnosis and ensure appropriate intervention.
**Gorter et al., 2022**	Canada	Cross-sectional study	Adolescents and young adults with cerebral palsy	Explore if demographics, social and clinical questionnaire scores, and cortisol levels in hair samples from adolescents and young adults with cerebral palsy are associated with higher scores on anxiety and/or depression questionnaires.	A high prevalence of mental health problems and co-occurring physical problems was found in adolescents and young adults with cerebral palsy. Integrating mental support into regular care for adolescents and young adults with cerebral palsy is recommended

**Hackworth et al., 2013**	Australia	Randomized controlled trial (protocol)	Adolescents with Type 1 diabetes	Present the research protocol for a study evaluating the efficacy of the Nothing Ventured Nothing Gained online adolescent and parenting intervention which aims to improve the mental health outcomes of adolescents with Type 1 diabetes.	NA
**Haller et al., 2022**	USA	Cross-sectional study	Children with Intellectual disability Children with autism spectrum disorder	Assess the unmet health care needs of children with intellectual disability compared with children with autism spectrum disorder and whether access to health insurance coverage is a contributing factor.	Children with intellectual disability were nearly four times more likely to have unmet medical needs than those with autism spectrum disorder, with similar patterns for hearing and mental health care. Those with both intellectual disability and autism spectrum disorder had higher overall unmet healthcare needs but fewer unmet medical care needs specifically.
**Halvorsen et al., 2023**	Norway	Systematic Review	Children and adolescents with intellectual disability	Review and evaluate the psychometric properties of instruments used to assess general mental health problems in children and adolescents with intellectual disability	Overall, the review indicated consistently better documentation of the reliability and validity of instruments designed for the intellectual disability population compared to instruments developed for the general child population.
**Hassiotis and Turk, 2012**	UK	Cross-sectional study	Adolescents with intellectual disability	Investigate the prevalence and predictors of mental health needs and service use in adolescents with intellectual disabilities.	Adolescents with intellectual disabilities often experience significant but under-recognized and untreated mental health problems, leading to functional impairment. Early identification and comprehensive assessment are essential to improve outcomes and reduce social exclusion.

**Hauptman and Barkoudah, 2022**	USA	Opinion article	Children with cerebral palsy	Highlight the role of neuropsychiatric symptoms in cerebral palsy and advocate for the integration of neuropsychiatry into multidisciplinary care.	Neuropsychiatric symptoms are common in individuals with cerebral palsy and interact complexly with neurological and psychosocial factors, often influencing overall functioning and treatment outcomes. Integrating neuropsychiatry into multidisciplinary care can improve diagnosis, management, and coordination of care for these individuals.
**Hobson et al., 2022**	UK	Qualitative design	Children with speech, language and communication needs	Explore parental experiences of seeking mental health support for children with speech, language and communication needs, particularly those with developmental language disorders.	Parents highlighted that relational, organizational, and professional knowledge factors shape access to mental health care for children with speech, language and communication needs. They reported that language difficulties often hinder recognition and appropriate support, with many services perceived as inaccessible or poorly adapted to their children's needs.
**Horridge, 2019**	UK	Opinion article	Children and adolescents with learning disabilities	Provide a structured overview of the identification, assessment, and management of children and adolescents with learning disabilities and to guide clinicians in delivering comprehensive, multidisciplinary care.	A structured, multidisciplinary approach that clearly identifies and documents all needs is essential to ensure equitable, high-quality care and improved outcomes for children and adolescents with learning disabilities.

**Hurley and Sovner, 1995**	USA	Case report	Adolescents with intellectual disability	Describe and examine clinical features of antisocial personality disorder (ASPD) in individuals with intellectual disability through a series of case studies.	The case reports suggest that ASPD can be identified in individuals with intellectual disability, although its presentation may differ and be complicated by cognitive and developmental factors. These findings highlight the importance of careful, adapted assessment to avoid misdiagnosis and to guide appropriate management.
**Jefferies et al., 2022**	Canada	Scoping Review	Children and adolescents with cerebral palsy, spina bifida and acquired brain injury	Identify key characteristics of psychological interventions being used to treat the mental health challenges of adolescents and adults with cerebral palsy, spina bifida, and childhood onset acquired brain injury.	There are a limited number of studies investigating psychological interventions for individuals with childhood onset acquired brain injury and cerebral palsy, and none for individuals with spina bifida. Further research into effective psychological interventions for these populations will improve mental health outcomes and transitional services.
**Jones et al., 2021**	Australia	Systematic Review	Children who stutter	Explore available measures for assessing the psychological impacts of stuttering in young school-age children and to examine their measurement properties.	The results highlight a lack of available measures in this domain and poor practices in developing and testing measurement instruments. To ensure that clinicians and researchers are equipped with sound measures to meet the mental health needs of this vulnerable population, further research to establish resources is needed.
**Kolaitis, 2008**	Greece	Narrative review	Children and adolescents with intellectual disability	Examine risk factors, types of psychopathologies, and their therapeutic management in young people with intellectual disabilities.	Children and adolescents with intellectual disabilities have complex and often unmet mental health needs, highlighting gaps in service provision. Early identification, improved access to care, and appropriate

					support for both individuals and families are essential to improve outcomes.
**Lal et al., 2022**	Canada	Scoping Review	Adolescents with motor impairment	Better understand the extent and nature of research activity on the topic of mental health problems in young people with childhood-onset physical disabilities.	Findings suggest the importance of developing integrated models of service delivery to identify and address the mental health needs of this population, and consensus on best practices for assessment and reporting rates of subclinical symptoms and psychiatric conditions.
**Du Feu and Fergusson, 2003**	UK	Opinion article	Children with hearing loss or deaf blindness	Review the impact of sensory impairments (hearing and visual) on mental health, including associated risk factors, developmental consequences, and implications for assessment and service provision.	Sensory impairments significantly affect mental health primarily through communication and social barriers, highlighting the need for early, multidisciplinary, and accessible care to improve outcomes.
**McCarthy, 2005**	UK	Narrative review	Adolescents with learning disabilities and behavioural problems	Review the prevalence, causes, assessment, and management of behavioural problems in adolescents with learning disabilities, with a focus on clinical practice.	Behavioural problems are highly prevalent and multifactorial in adolescents with learning disabilities, requiring comprehensive assessment and coordinated, multidisciplinary, and multimodal management.
**Miniscalco et al., 2006**	Sweden	Cohort Study	Children with speech, language and communication needs	Examine prospectively the neuropsychiatric and neurodevelopmental outcomes at school age of children identified with early language delay at around 30 months.	Early language delay strongly predicts later neurodevelopmental disorders, with children screening positive before age 3 at high risk of neuropsychiatric or neurodevelopmental disorders at age 7 years.

**Mulvale et al., 2019**	Canada	Narrative review	Children and adolescents with learning and intellectual disability	Review literature about child and adolescent mental health services (CAMHS) and adult mental health services (AMHS) care philosophies and their influence on transitions.	Findings highlight clear differences in care philosophies between CAMHS, which adopt a developmental and family-oriented approach, and AMHS, which emphasize diagnosis, autonomy, and individual responsibility. Greater awareness of these differences, along with improved collaboration, may support more coordinated care for young people transitioning between services.
**Pratt and Patel, 2007**	USA	Narrative review	Children and adolescents with learning disabilities	Provide a comprehensive overview of learning disorders in children and adolescents, including their definition, causes, clinical presentation, assessment, and management.	Learning disorders are complex, multifactorial conditions that require early identification and multidisciplinary, individualized interventions to improve academic, social, and long-term outcomes.
**Sequeira and Hollins, 2003**	UK	Narrative review	Children and adolescents with learning disabilities	Critically review the literature concerning the psychological reactions of people with learning disabilities to sexual abuse	Several studies suggest that, following sexual abuse, people with learning disabilities may experience a range of psychopathology similar to that experienced by adults and children in the general population. However, because of methodological limitations, these results are not conclusive.
**Singh, 2009**	UK	Narrative review	Children and adolescents with learning and intellectual disability	To synthesise current research and policy literature on transition to determine the magnitude of the problem, barriers at the interface between child and adolescent mental health services (CAMHS) and adult mental health services (AMHS) and outcomes of poor transition.	Poorly managed transitions can disrupt continuity of care, increase disengagement, and worsen outcomes, particularly for young people with neurodevelopmental and complex needs. Strengthening the evidence base on transition processes is essential to develop effective interventions and reduce care gaps.

**Sundheim and Voeller, 2004**	USA	Narrative review	Children and adolescents with learning disabilities	Examine the relationships between language and learning disorders and associated psychiatric conditions, as well as their clinical implications and management.	Language and learning disorders are strongly associated with early and persistent psychiatric comorbidities, highlighting the need for early identification and multidisciplinary intervention to improve developmental outcomes.
**Teverovsky et al., 2009**	USA	Cross-sectional study	Children with speech, language and communication needs	Describe the complex array of functional problems in children diagnosed by their treating speech/language pathologist with Childhood Apraxia of Speech (CAS), a poorly understood, severe speech sound disorder.	The ICF-CY provided a systematic approach for describing and categorizing functional problems in children with CAS. The identified factors should guide the multidisciplinary team in conducting comprehensive evaluations, rehabilitation, and long-term follow-up of children with CAS.
**Thomsen et al., 2023**	Denmark	Cross-sectional study	Children and adolescents with profound and multiple disabilities	Examine whether wellbeing, health behavior, and youth life among young people with co-occurrence of physical-mental conditions differs from young people with exclusively physical or mental conditions	Young people with physical-mental multimorbidity had higher odds for challenges and low wellbeing and life satisfaction. This is an especially vulnerable group and systematic screening for multimorbidity, and psychosocial wellbeing is needed in all healthcare settings.
**Tremblay et al., 2023**	Canada	Scoping Review	Adolescents with motor impairment	Synthesize scientific literature related to the organization and delivery of services for youth with co-occurring childhood-onset physical disabilities (e.g., cerebral palsy, spina bifida) and mental health problems (e.g., depression, anxiety).	This review identifies key principles for organizing and delivering collaborative mental health care for children with complex needs, emphasizing coordination, training, and integrated service models. However, further research is needed to develop and evaluate effective collaborative care models.

**Trollor, 2018**	Australia	Opinion article	Children and adolescents with intellectual disability	Highlight the gap between disability support systems and mental health care for people with intellectual disabilities and advocate for urgent improvements in service provision and system integration.	Despite high mental health needs, people with intellectual disability face inadequate, poorly coordinated, and insufficiently trained services, requiring urgent systemic, multidisciplinary, and policy-level action.
**Vedi and Bernard, 2012**	UK	Narrative review	Children and adolescents with learning disabilities	Provide an update on the mental health needs of children and adolescents with learning disabilities, by examining salient studies published predominantly in the last 12–18 months.	Although there is improved knowledge of the rates of mental health disorders in young people with learning disabilities, in clinical practice these mental health needs continue to be underrecognized and untreated.
**Vohra et al., 2014**	USA	Cross-sectional study	Parents of children and adolescents with autism spectrum disorders<br><br>Parents of children and adolescents with other developmental disabilities	Examine perceived access to services, quality of care, and family impact reported by caregivers of children aged 3–17 years with autism spectrum disorders, as compared to caregivers of children with other developmental disabilities and other mental health conditions.	Caregivers of children with autism spectrum disorders were significantly more likely to report difficulty using services, lack of source of care, inadequate insurance coverage, lack of shared decision making and care coordination, and adverse family impact as compared to caregivers of children with developmental disabilities, mental health conditions, or both.
**Whittle et al., 2018**	Australia	Systematic review	Children and adolescents with intellectual disability	Identify the current literature pertaining to the barriers and facilitators to access to mental health services for people with an intellectual disability.	Access to mental health care for people with intellectual disability is hindered by organizational barriers, limited and low-quality services, and gaps in professional knowledge, while collaboration and training act as key facilitators. However, significant evidence gaps—particularly regarding lived experiences—highlight the need for further research and evaluation to improve access and care quality.

**Whitney et al., 2019**	USA	Cross-sectional study	Children and adolescents with intellectual disability	Determine factors associated with depression and anxiety problems in children with intellectual disabilities.	The present study identified both treatable and modifiable, as well as unmodifiable, factors associated with depression and/or anxiety problems in children with intellectual disabilities.
**Xiong et al., 2022**	Canada	Cross-sectional study	Parents of children with intellectual disability	Develop and validate a scale for measuring barriers to accessing mental healthcare.	Financial barriers and limited service availability were identified as key obstacles to mental healthcare access for parents of children with intellectual disability; a scale measuring these barriers was developed and validated.

Note: The term 'learning disabilities' in UK literature usually corresponds to 'intellectual disabilities' in international nomenclature.
